# Automated structure and flow measurement — a promising tool in nailfold capillaroscopy

**DOI:** 10.1016/j.mvr.2018.03.016

**Published:** 2018-07

**Authors:** Michael Berks, Graham Dinsdale, Andrea Murray, Tonia Moore, Joanne Manning, Chris Taylor, Ariane L. Herrick

**Affiliations:** a Centre for Imaging Sciences, Division of Informatics, Imaging & Data Sciences, The University of Manchester, Manchester, UK; b Division of Musculoskeletal & Dermatological Sciences, The University of Manchester, Manchester Academic Health Science Centre, Manchester, UK; c Salford Royal NHS Foundation Trust, Salford, UK; d NIHR Manchester Musculoskeletal Biomedical Research Centre, Manchester University NHS Foundation Trust, Manchester Academic Health Science Centre, Manchester, UK

**Keywords:** Systemic sclerosis, Nailfold capillaroscopy, Red blood cell velocity, Microcirculation

## Abstract

**Objectives:**

Despite increasing interest in nailfold capillaroscopy, objective measures of capillary structure and blood flow have been little studied. We aimed to test the hypothesis that structural measurements, capillary flow, and a combined measure have the predictive power to separate patients with systemic sclerosis (SSc) from those with primary Raynaud's phenomenon (PRP) and healthy controls (HC).

**Methods:**

50 patients with SSc, 12 with PRP, and 50 HC were imaged using a novel capillaroscopy system that generates high-quality nailfold images and provides fully-automated measurements of capillary structure and blood flow (capillary density, mean width, maximum width, shape score, derangement and mean flow velocity). Population statistics summarise the differences between the three groups. Areas under ROC curves (A_Z_) were used to measure classification accuracy when assigning individuals to SSc and HC/PRP groups.

**Results:**

Statistically significant differences in group means were found between patients with SSc and both HC and patients with PRP, for all measurements, e.g. mean width (μm) ± SE: 15.0 ± 0.71, 12.7 ± 0.74 and 11.8 ± 0.23 for SSc, PRP and HC respectively. Combining the five structural measurements gave better classification (A_Z_ = 0.919 ± 0.026) than the best single measurement (mean width, A_Z_ = 0.874 ± 0.043), whilst adding flow further improved classification (A_Z_ = 0.930 ± 0.024).

**Conclusions:**

Structural and blood flow measurements are both able to distinguish patients with SSc from those with PRP/HC. Importantly, these hold promise as clinical trial outcome measures for treatments aimed at improving finger blood flow or microvascular remodelling.

## Highlights

• Novel system allows rapid capture of high-quality capillary structure and flow images.
• Nailfold capillary structure and flow can be measured automatically.
• Combining capillary structure and flow strongly discriminates disease from normality.
• Flow, significantly reduced in SSc, shows potential as a functional outcome measure.

## 1 Introduction

The value of nailfold capillaroscopy in the early diagnosis of systemic sclerosis (SSc) has long been recognised. At the nailfold, capillaries run parallel to rather than perpendicular to the skin surface, and can be easily seen when magnified. Characteristic abnormalities in patients with SSc include dilated capillary loops (including giant capillaries), distortion of the normal capillary architecture, areas of avascularity, and areas of haemorrhage (Maricq and LeRoy, 1973; Herrick and Cutolo, 2010; Smith et al., 2016). Because Raynaud's phenomenon (RP) is the most common presenting symptom of SSc, nailfold capillaroscopy is a key investigation in patients presenting with RP: abnormal nailfold capillaries allow early diagnosis of SSc (LeRoy and Medsger, 2001; Koenig et al., 2008; Matucci-Cerinic et al., 2009; Avouac et al., 2011) and are included in the 2013 American College of Rheumatology (ACR)/European League against Rheumatism (EULAR) Classification Criteria for SSc (Van den Hoogen et al., 2013).

Most clinical applications and research studies concerning nailfold capillaroscopy relate to imaging of microvascular structure. However, capillary flow (one aspect of function), can also be assessed, potentially providing additional insights into pathogenesis and measurement of the SSc disease process. Measuring capillary blood flow is challenging, and previous work estimating capillary flow in nailfold capillaroscopy videos has estimated flow only at manually selected points or vessels, leading to subjectivity – if only a small number of vessels are selected then blood flow in these may be unrepresentative of the whole nailfold (Shih et al., 2011; Mugii et al., 2009). To address the inherent challenges, we have developed a system for measuring both structure and flow fully-automatically in all visible capillaries across the whole nailfold. The aim of this study was to apply this novel capillaroscopy system, incorporating flow measurements, in a cross-sectional study of patients with SSc, patients with primary (idiopathic) Raynaud's phenomenon (PRP), and healthy control subjects. Our hypothesis was that a combination of capillary flow and structural measurements would be better able to discriminate between patients with SSc and those with PRP (or healthy controls), than any single measurement.

## 2 Patients

112 subjects were recruited into the study after signing informed consent: the study was approved by the NRES Committee North West - Greater Manchester East ethics committee (study reference: 14/NW/1436). Fifty were patients with SSc [45 (90%) female, 4 (8%) current smokers, median age (range) 60 (22–83) years, median duration (range) of RP 21 (1–67) years, median duration (range) of SSc since first non-RP clinical manifestation 12 (1−30) years, 8 (16%) had a history of hypertension and two (4%) had diabetes]. Twelve had PRP [10 (83%) female, 0 (0%) current smokers, median age (range) 47 (24–68) years, median duration (range) of RP 15 (1–60) years, one (8%) had a history of hypertension]. Fifty were healthy controls (HC) [33 (66%) female, 3 (6%) current smokers, median age (range) 51 (27–85) years]. At the time of imaging, 33/50 (66%) of the patients with SSc, and 3/12 (25%) of the patients with PRP were taking vasodilatory medication.

## 3 Methods

### 3.1 Nailfold capillaroscopy system

**Fig. 1:**
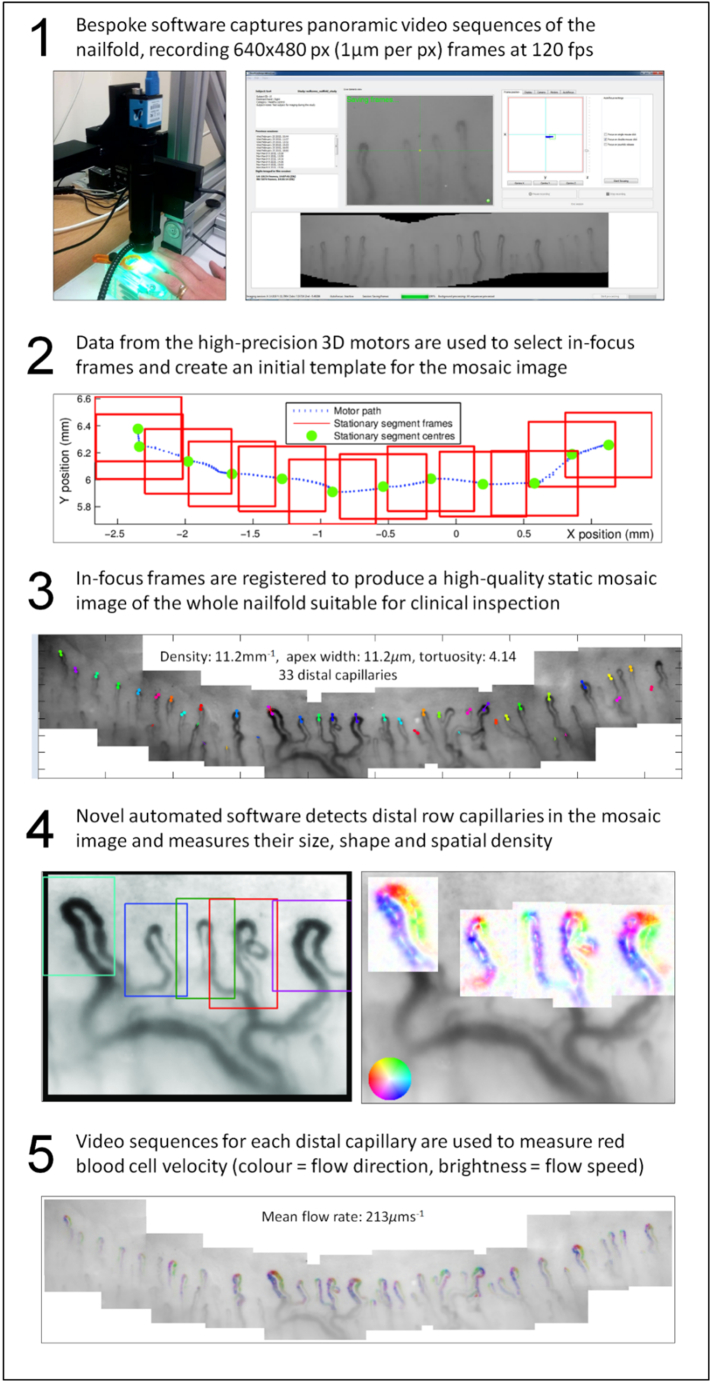
Image capture, vessel detection, and flow sequence estimation. All video image frames are 640 × 480 pixels with a resolution of 1 μm per pixel. Frames are stitched in software to produce mosaic images across the whole nailfold. Panels are as follows: (1) An overview of the microscope system and capture software interface; (2) Diagram of motor positions and tracking data used to inform frame stitching and the mosaic creation process; (3) A fully-registered mosaic image with automatically detected vessels highlighted, counted and measured; (4) Individual regions from the mosaic in (3), showing vessel region detection (left) and vessel path orientation (right); (5) Structural mosaic from (3) overlaid with false colour flow information extracted from vessels using optical flow techniques.

The nailfold capillaroscopy system which we have developed is described fully elsewhere (Berks et al., 2014; Berks et al., 2016). In brief, the system uses a high frame rate (120 frames per second) camera to capture video sequences which allow measurement of red blood cell velocity in individual capillaries. The camera is mounted on a software-controlled 3-axis motorised stage which allows sequences to be captured more quickly (approximately 1 min per finger) than with conventional manually-adjusted microscope systems. Novel software then generates high-quality static nailfold capillary image mosaics (Fig. 1, and see Supplementary video 1) and subsequently makes fully-automated measurements of capillaroscopy structure and flow (Berks et al., 2014; Murray et al., 2011). The image processing and automated measurement take 1–2 min per nailfold on a standard desktop computer. Since there is no alternative method to measure flow velocity for individual capillaries, we validated the flow measurement algorithm using realistic software phantom data (Tresadern et al., 2013)

### 3.2 Imaging protocol

Participants were acclimatised in a temperature-controlled laboratory for 20 min prior to imaging. For each participant, video sequences were taken from all 10 digits (where available). These sequences were then used to generate a total of 1104 static nailfold mosaic images. A total of 16 digits were not imaged; reasons included the presence of obscuring dressings because of ulcers/calcinosis (3 digits from 1 participant), amputations (5 digits from 2 participants), severe contractures (7 digits from 1 participant), and equipment failure (1 digit from 1 participant).

### 3.3 Automated image analysis

For each mosaic, together with its corresponding video sequence, automated software was used to compute measures of capillary density, mean and maximum width, shape, derangement and mean flow velocity. To generate these parameters the software first detects each distal row capillary and estimates the location of its apex and the path the capillary follows along the arterial and venous limbs. The width and orientation at each point along the capillary path up to a distance of 100 μm from the apex are then estimated, and used to compute the capillary's average width, principal orientation (the mean of the path orientations) and shape score (the dispersion statistic (Mardia, 2000) of the path orientations – this varies between 0 and 1 and will be low for capillaries with highly tortuous, abnormal shapes and high for normal hairpin shaped capillaries). The software also estimates mean blood flow velocity along each capillary path, using all video frames in which the capillary apex was present.

These capillary-level parameters were used to produce the following image-level parameters:

a. Capillary density (number of capillary apices per millimetre, measured from the left-most to the right-most capillaries).
b. Mean width (the mean of the individual capillary widths).
c. Max width (the largest of the individual capillary widths).
d. Shape score (the mean of the individual capillaries shape scores).
e. Derangement score (the dispersion statistic of the principal orientations of each capillary – as with the shape score, this varies between 0 and 1, and will be low in nailfolds with highly irregular capillary structure and high where all capillaries ‘line-up’ in a common direction).
f. Mean flow velocity (the mean of the individual capillary flow measures).

Finally, these six nailfold-level measurements were averaged across all imaged digits to produce corresponding participant-level parameters.

### 3.4 Statistical analysis

For each parameter one-way ANOVA and Tukey's range test were computed to check for group differences. The area under ROC curve (A_Z_) was used to measure separation between SSc and PRP/HC groups. Healthy controls and patients with PRP were combined for this analysis because it is generally considered that in patients with PRP, the nailfold capillaries are normal (this is one of the defining characteristics of PRP (LeRoy and Medsger, 1992; Maverakis et al., 2014)) and in our analysis (see below) results were similar between healthy controls and patients with PRP for all parameters except maximum width (which has previously been shown to be increased in patients with PRP compared to healthy controls (Bukhari et al., 1996)). We combined the individual parameters in a logistic regression model using HC/PRP vs SSc as a binary output variable, first using only the structural measures, and then including flow. Stepwise regression was used to add/remove terms from an initial linear model fit, and in both cases max width (highly correlated with mean width) and derangement (highly correlated with shape) were discarded. In the latter model flow was retained suggesting it provides additional independent information to the structural measures. To estimate model performance we applied leave-one-out cross-validation to obtain unbiased predictions for each subject.

## 4 Results

**Fig. 2:**
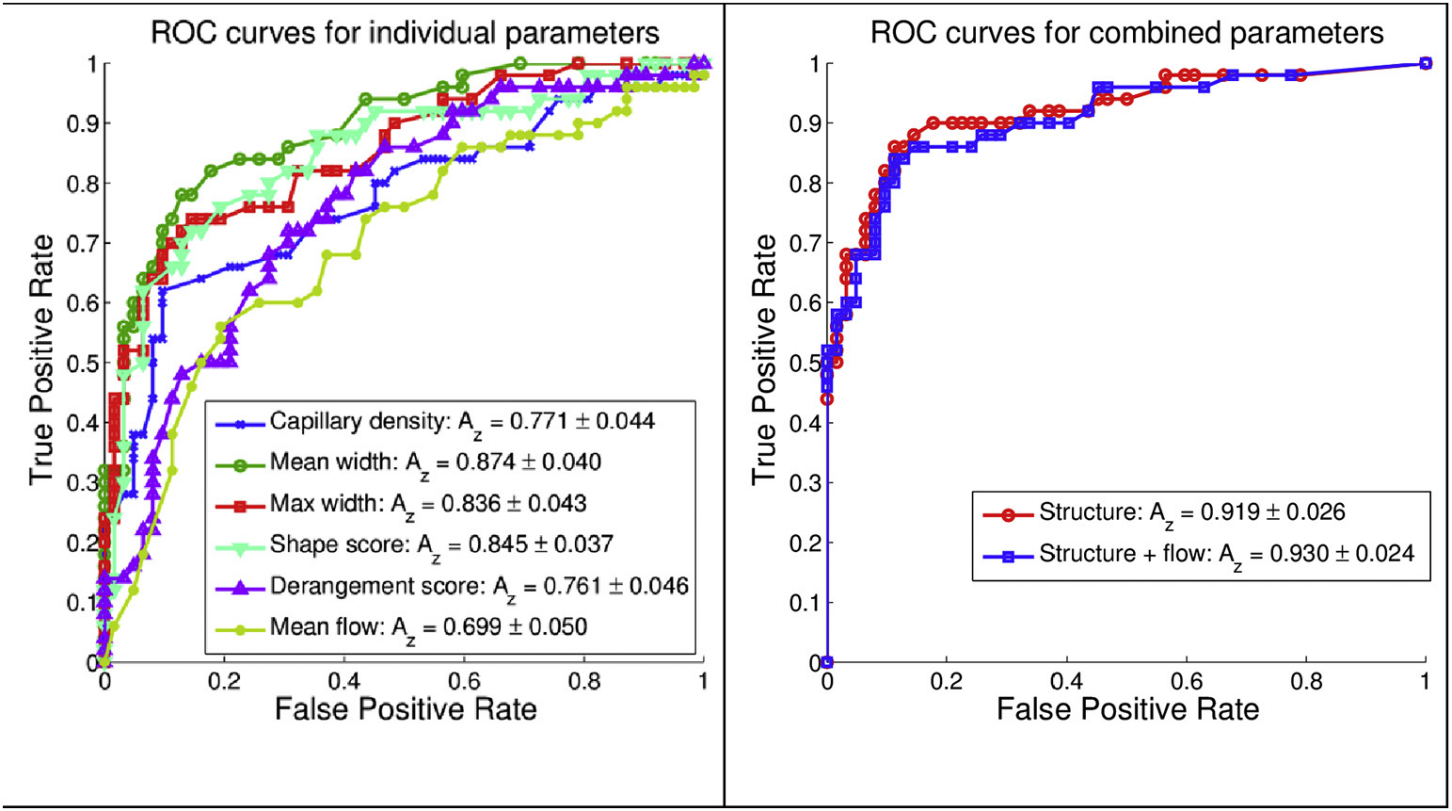
ROC curves for single capillary parameters (a: left), and combined models (b: right). Area under the curves (A_Z_) and standard errors are shown for each colour-coded curve. (For interpretation of the references to colour in this figure legend, the reader is referred to the web version of this article.)

Group means for the six measured parameters (5 structure + flow) are shown in Table 1. Additionally in Table 1 are the A_Z_ values for the individual parameters, and the two regression models (structure alone, and structure with flow). Group means for patients with SSc were statistically significantly different from both healthy controls and patients with PRP for all parameters, including blood flow velocity. Fig. 2a shows ROC curves and A_Z_ values for predicting SSc (positive) versus the combined healthy control/PRP group (negative) for each individual parameter. Fig. 2b shows ROC curves for: (1) the five structural parameters combined (ROC A_Z_ = 0.919 ± 0.026), which was greater than the best single parameter in Fig. 2a (mean width, ROC A_Z_ = 0.874 ± 0.043); and (2) the five structural parameters plus flow (ROC A_Z_ = 0.930 ± 0.024).

**Table 1:** Group means and standard errors and ROC A_Z_ values for each capillary measure. ^#^, †, ‡ denote significant pair-wise differences for PRP vs HC, SSc vs HC, and SSc vs PRP respectively.

Parameter	Subject group means (±s.e.)			ROC Az
	HC (n = 50)	PRP (n = 12)	SSc (n = 50)	HC,PRP v SSc
Capillary density (mm^−1^)	6.73 ± 0.34	7.28 ± 0.54	5.52 ± 0.42 †‡	0.771 ± 0.04
Mean width (μm)	11.8 ± 0.23	12.7 ± 0.74	15 ± 0.71 †‡	0.874 ± 0.04
Max width (μm)	16.5 ± 0.91	21.4 ± 2.94^#^	27.4 ± 2.38 †‡	0.836 ± 0.04
Shape score (∈ [0, 1])	0.312 ± 0.02	0.325 ± 0.03	0.249 ± 0.01 †‡	0.845 ± 0.04
Derangement score (∈ [0, 1])	0.649 ± 0.03	0.689 ± 0.05	0.54 ± 0.03 †‡	0.761 ± 0.05
Mean flow velocity (mm s^−1^)	0.311 ± 0.05	0.383 ± 0.10	0.235 ± 0.04 †‡	0.726 ± 0.05
Combined structure parameters	–	–	–	0.919 ± 0.026
Structure and flow parameters	–	–	–	0.930 ± 0.024

## 5 Discussion

We have confirmed that capillaroscopic parameters allow differentiation of patients with SSc from those with PRP/HC. Key new findings are that (1) blood velocity provides discrimination comparable with individual structural parameters, (2) combining structural parameters, measured automatically at high speed (approximately 1 min per nailfold), improved differentiation (compared to individual parameters); (3) blood velocity results, although preliminary, provide complementary information for distinguishing SSc from PRP/HC and combining velocity with structural measurements further improved discrimination performance.

The ability to measure capillary flow velocity objectively is a very major step forward and potentially opens up a new era for nailfold capillaroscopy as a non-invasive investigative tool. In recent years there has been a huge increase in application of nailfold capillaroscopy by rheumatologists (in part fuelled by inclusion of abnormal capillaroscopy in the ACR/EULAR criteria (Van den Hoogen et al., 2013)), evidenced by increasing numbers of research publications and EULAR and British Society for Rheumatology sponsored training courses. *Structural* measurements will always be those most relevant to practicing rheumatologists, the major application of capillaroscopy being in the assessment of the patient presenting with RP. However, for the clinical researcher, it is important to point out that structural abnormalities are likely to develop and progress relatively slowly: probably too slowly to be useful as outcome measures in most clinical trials, although this is an area of current research and increasingly investigators are including capillaroscopy in studies of treatment response (Moore et al., 2007; Guiducci et al., 2012; Cutolo et al., 2013). Conversely, capillary blood flow is likely to change rapidly in response to, for example, vasodilator drug treatment, and could therefore be an outcome measure especially in early phase, proof-of-concept trials as well as in studies of pathophysiology of SSc and other rheumatological conditions in which the microvasculature is thought to contribute to pathogenesis. Relevant to this, Mugii et al. (2009), as well as reporting reduced red blood cell velocity in nailfold capillaries from patients with SSc compared to healthy controls, reported that velocity increased in seven patients with SSc after treatment with alprostadil, underscoring how red cell blood velocity is likely to be much more sensitive to change than structural capillaroscopic parameters. Increased nailfold red blood cell velocity in response to vasoactive treatments has also been reported in other conditions, for example to antioxidants (in the context of smoking) (Henriksson et al., 2011) and to moxonidine (in the context of hypertension) (Martina et al., 1998). By allowing rapid measurement of velocity, averaged across the whole nailfold, our automated methodology brings the potential of sensitive, accurate, and rapid measurement for application in early phase clinical trials. A limitation of our study was that all patients with SSc had well established SSc, with a median disease duration of 12 years, and it will be important to test ability to discriminate between patients with PRP and those presenting with early SSc, so as to obtain results which are generalisable to those patients presenting with RP in everyday practice. Therefore larger scale studies including patients with early disease, and examining patients prospectively over time, are now required. Also, to assess the sensitivity of flow measures, we are planning studies including different dynamic challenges, to test the ability of our system to measure flow changes in response to (for example) temperature changes and occlusion. Future studies could also incorporate comparison of flow measurements to measurements obtained using other physiological measurement techniques to validate/calibrate our flow velocity measures, for example thermography which measures surface temperature or laser Doppler imaging which directly measures blood cell movement (though not for individual capillaries).

In conclusion, we have developed a state-of-the-art nailfold capillaroscopy system which measures nailfold capillary structure and blood flow automatically, with a substantial benefit in terms of operator time. Adding blood flow to structural measures may (with further refinement) help distinguish patients with SSc from those with PRP, and holds promise as an outcome measure in clinical trials of treatment aimed at either improving finger blood flow or remodelling the microvasculature. Finally, our novel system could be modified to assess, non-invasively, other aspects of capillary function, for example oxygenation (using multispectral imaging) and oxidative stress (using ultraviolet-induced fluorescence).

The following is the supplementary data related to this article.

Supplementary video 1 Video illustrating and describing capillaroscopy image acquisition and analysis. Supplementary video 1

## Funding statement

This work was funded by the Wellcome Trust (09342/Z/10/Z).

## Declarations of interest

None.
